# Serial Magnetic Resonance Imaging in Creutzfeldt-Jakob Disease: a Case Report and Literature Review

**DOI:** 10.7759/cureus.1095

**Published:** 2017-03-14

**Authors:** Ahmed H Qavi, Tasnim F Imran, Zachariah Hasan, Fariha Ilyas, Usman Ghani, Salman Assad, Shabih Hasan

**Affiliations:** 1 Department of Medicine, Montefiore New Rochelle Hospital, Albert Einstein College of Medicine, New Rochelle, NY, USA; 2 Department of Medicine, Brigham and Women’s Hospital, Harvard Medical School, Boston, MA, United States; 3 Department of Medicine, Eastern Virginia Medical School, Norfolk, VA, United States; 4 Department of Medicine, University of Texas at Austin, Dell Medical School, Austin, TX, USA; 5 Department of Medicine, Shifa International Hospital, Islamabad, Pakistan; 6 Department of Medicine, Shifa Tameer-e-Millat University, Islamabad, Pakistan; 7 Department of Neurology, Inova Fairfax Hospital, Falls Church, VA, United States

**Keywords:** creutzfeldt-jakob disease (cjd), degenerative disease, old age

## Abstract

Creutzfeldt-Jakob disease (CJD) is a rare, degenerative, invariably fatal brain disorder. CJD usually appears in later life and runs a rapid course. Typically, the onset of symptoms occurs about age 60 and about 90% of individuals die within one year. We report a case of 67-year-old male presented with progressive aphasia, confusion, dysphagia and inability to carry out activities of daily life (ADLs) over a period of three to four weeks. The patient had past medical history of chronic atrial fibrillation and hypertension. Prior to admission, the patient was treated for ischemic stroke of left basal ganglia but continued to have worsening encephalopathy. The spinal tap revealed a 14-3-3 protein level of thirteen times the upper limit of normal; electroencephalogram (EEG) showed a diffuse slowing of the background and periodic sharp waves with greater involvement of the left hemisphere. Diffusion-weighted imaging (DWI) magnetic resonance imaging (MRI) at the time of admission showed extensive signal abnormality in the basal ganglia bilaterally and in the cerebral cortex bilaterally, particularly over the left cerebral hemisphere. The persistence of the MRI findings over several weeks was concerning for spongiform encephalopathy. The probable diagnosis of Creutzfeldt-Jakob disease was made based on these imaging findings taken together with the patient’s clinical signs and symptoms of a rapidly progressive encephalopathy. The patient was able to have some quality time with his family as the diagnosis was made earlier than perhaps otherwise and expired peacefully after comfort care measures were chosen. Serial MRI may serve as a clue to the early diagnosis of CJD and potentially provide a better quality of life for the patients.

## Introduction

Creutzfeldt-Jakob Disease (CJD) is a type of human prion disease. It has been suggested to be the most common cause of rapidly progressive neurodegenerative dementia. The diagnosis of CJD is generally made with the patient’s clinical manifestations and markers such as an elevated cerebrospinal fluid (CSF) protein 14-3-3 level [1]. Magnetic resonance imaging (MRI) is a valuable tool in the diagnosis of CJD but is currently not included in its diagnostic criteria. An MRI of a CJD patient usually demonstrates hyperintense signal changes in the striatum or thalamus on T2-weighted images. It may also show lesions in the periventricular white matter and diffuse cortical atrophy [2-3]. Utilization of serial MRI over a period of time for diagnosis of CJD have not been reported widely in the literature. We report a case in which serial MR images aided in the early diagnosis and follow-up of CJD. Informed consent statement was obtained for this study.

## Case presentation

Our patient is a 67-year-old male with a history of hypertension, hyperlipidemia, atrial fibrillation, left basal ganglia infarct and mitral valve prolapse who developed periods of altered mental status and inability to concentrate a month prior to presentation. One month prior to admission, he was treated for ischemic stroke, started on aspirin and discharged to a rehabilitation center for assistance with ambulation and activities of daily life (ADLs). The patient was subsequently readmitted this time due to worsening confusion and encephalopathy. He denied any history of travel, contact with wild animals or ticks or similar symptoms amongst close contacts.

On physical examination, he was found to be mildly cachectic, appeared older than stated age, confused and non-communicative. He had a low-grade fever, most likely due to aspiration or atelectasis. He was alert, oriented to person only, had severe expressive aphasia and difficulty in counting fingers. No gross paresis was appreciated and extraocular movements were intact. Cranial nerves II to XII were grossly intact. The patient had some cogwheel rigidity of his arms and legs, with loss of primitive reflexes, and flexor plantar reflexes bilaterally. A foot twitch was periodically noted. On motor examination, the tone was normal, strength was 5/5 in all four extremities, reflexes were 1+ symmetrical. Coordination was intact with no signs of dysmetria or dysdiadochokinesia. Blood and urine cultures were normal. Vitamin B12 level was 329 ng/L. CSF analysis revealed normal cells and glucose and was negative for herpes simplex virus (HSV one and HSV two). CSF protein 14-3-3 was elevated at thirteen times the upper limit of normal. The patient was started on Piperacillin-Tazobactam and Vancomycin empirically for suspected pneumonia and aspirin and low-dose heparin for secondary prevention of stroke. 

**Figure 1:**
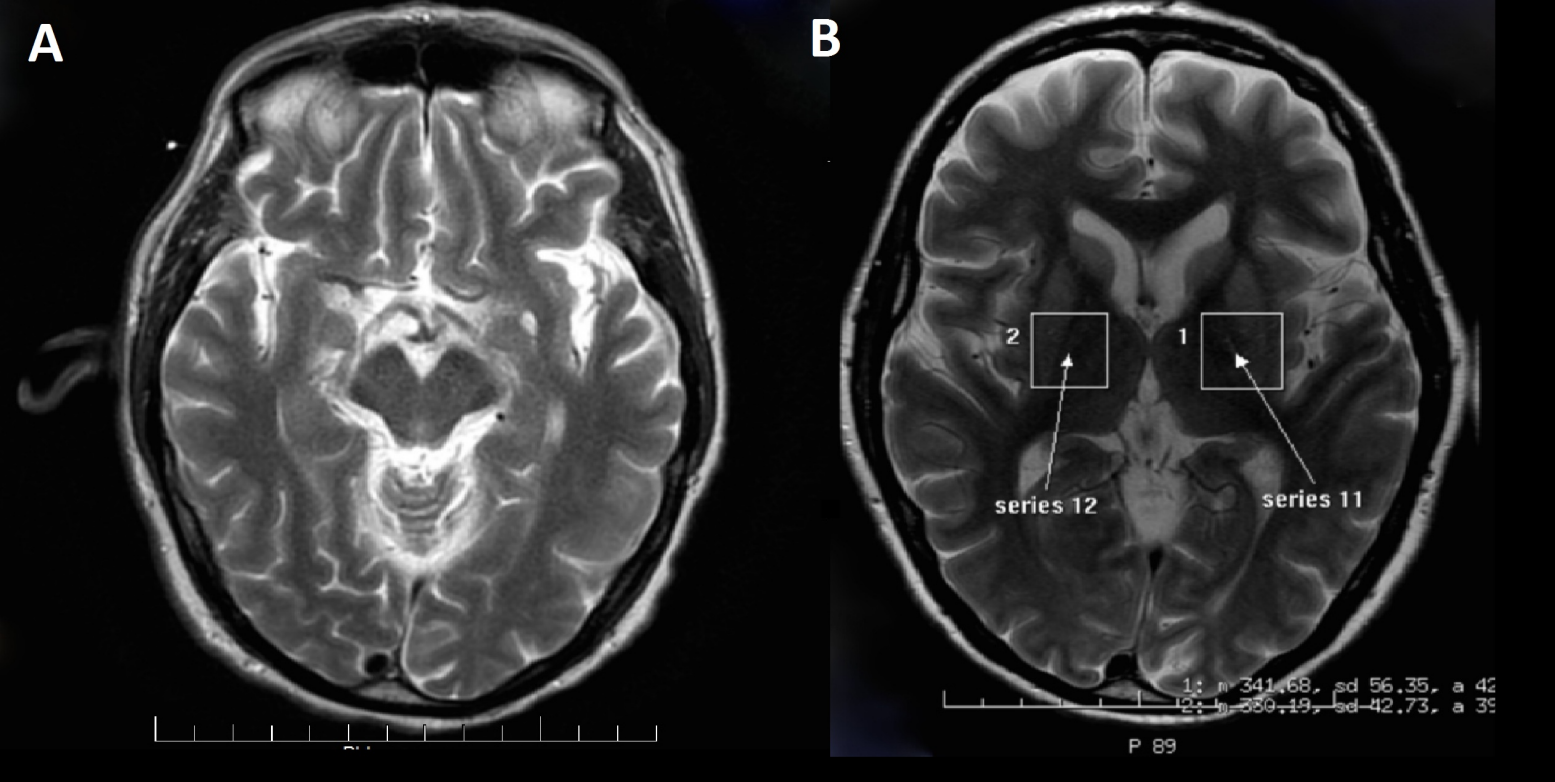
Diffusion weighted imaging (DWI) MRI [A, B] Shows extensive signal abnormality in the bilateral basal ganglia and cerebral cortex

Computed tomography (CT) scan was negative for any acute pathology. Electroencephalography (EEG) showed a diffuse slowing of the background with greater involvement of the left hemisphere and left frontocentral blunted sharp transients. There were periodic generalized discharges. The focal findings and sharp activity were more prominent than a previous EEG done earlier that month. The EEG findings indicated diffuse cerebral dysfunction with greater involvement of the left hemisphere. Diffusion-weighted imaging (DWI) MRI at the time of admission showed extensive signal abnormality in the basal ganglia bilaterally and in the cerebral cortex bilaterally, particularly over the left cerebral hemisphere (Figure 1).

**Figure 2:**
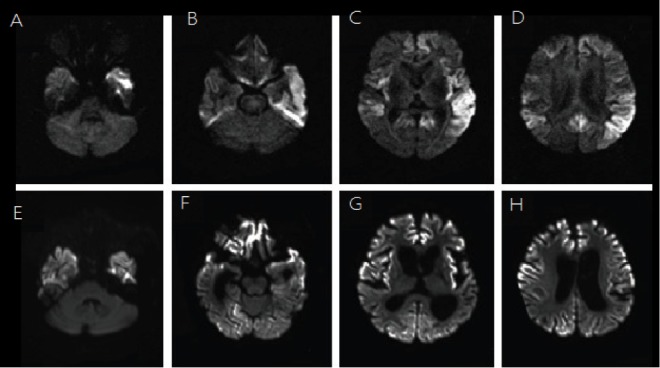
MRI brain [A-H] Shows persistent diffusion abnormality involving the lentiform nucleus on the left, with mild abnormality involving the lentiform nucleus on the right and diffusion abnormality in the cortex of the cerebral hemispheres, left greater than right

The persistence of the MRI findings was concerning for spongiform encephalopathy. Taken together with the clinical findings of progressive encephalopathy, the patient was diagnosed with Creutzfeldt-Jakob disease (Figure 2).

The patient was extubated from mechanical ventilation and provided with comfort measures as per his family’s wishes. Shortly thereafter, he expired with his family at the bedside.

## Discussion

CJD is the most frequent of the human prion diseases, although quite rare. Approximately one case of CJD occurs per 1,000,000 populations per year. The mean age onset of CJD is between 57 and 62 years with no gender predilection [4]. Patients typically present with rapidly progressive mental deterioration and myoclonus. This could manifest as dementia, behavioral and personality changes and cortical deficits. Memory, concentration and impaired judgment are early manifestations along with mood changes, apathy, and depression. CJD should be suspected in any patient with a rapidly progressive dementia and myoclonus [5]. In this report, our patient presented with rapidly progressive dementia and inability to concentrate. 

MRI is a helpful imaging technique for CJD. It usually shows an abnormal signal in the putamen and head of the caudate. Early CJD is characterized by an increased diffusion-weighted imaging (DWI) signal in the cortex or deep gray matter. Intermediate CJD is recognized by greater symmetrical involvement and progression of lesions to involve the putamen. Late CJD is characterized by generalized atrophy and ventricular dilatation [5]. The role of serial MRI has not been well established as a diagnostic tool for CJD. However, evidence suggests it may not only help in the diagnosis but also aid in monitoring the disease [6]. In 2011, Kono, et al. reported that serial MRI in a CJD patient could show the progression of abnormal lesions starting from the left cerebral cortex, head of caudate and the putamen to the right cerebral cortex. They eventually spread to the occipital lobes in the terminal stage [7]. Others have commented on the advancement of abnormalities from the basal ganglia to cerebral gray and white matter as demonstrated in a follow-up MRI. Oppenheim, et al. pointed out the correlation of the patient’s clinical and pathological progression to the hyperintense signal MR abnormalities [8]. The study reported that as the patient declined to a near vegetative state, the abnormal hyperintense lesions were no longer clearly apparent. This was thought to occur because terminal pathological cortical changes in the form of fibrillary gliosis and gross neuronal loss had taken place [8]. Other case reports have also confirmed the use of serial MRI to identify CJD and its progression [9]. In our patient, the persistence of diffusion abnormality involving the lentiform nucleus and cerebral cortex with expansion was a high-yield clue in the diagnosis of CJD as observed in our patient.

Periodic sharp wave complexes on EEG have a high specificity for CJD. The 14-3-3 protein in CSF has been found to be a sensitive and specific marker for sporadic CJD (sCJD). It is used as an adjunctive test along with other findings for the diagnosis of prion diseases [10]. A brain biopsy is the gold standard test to make a definitive diagnosis. However, it is often unnecessary. A clinical presentation with supporting findings on MRI, EEG, and CSF are usually sufficient to exclude other causes and make a probable diagnosis of CJD. This case illustrates a clinical presentation and imaging findings consistent with the diagnosis of probable CJD. It is most likely that this patient had sCJD. His clinical presentation of rapidly progressive deterioration of mental status, along with persistent MRI and EEG findings, markedly elevated 14-3-3 protein levels, and exclusion of metabolic or toxic derangements suggest the diagnosis of CJD. A high index of suspicion is required to make the diagnosis, as patients may not have a typical presentation. Our patient had a stroke, which initially obscured the diagnosis, but as he continued to worsen, further workup was initiated urgently. These patients continue to deteriorate with worsening quality of life over months. Thus, an earlier diagnosis would alleviate discomfort and tension for patients and family members. Serial MRI may be a particularly useful non-invasive modality in aiding with the diagnosis, as with our patient. The persistence of MRI findings involving the lentiform nucleus and expansion in the cerebral cortex, in this case, were suggestive of a spongiform encephalopathy. The patient was able to have some quality time with his family as the diagnosis was made earlier than perhaps otherwise and expired peacefully after comfort care measures were chosen.

## Conclusions

The patient was able to have some quality time with his family as the diagnosis was made earlier than perhaps otherwise and expired peacefully after comfort care measures were chosen. Serial MRI may serve as a clue to the early diagnosis of CJD and potentially provide a better quality of life for the patients.
